# Path creation as a discursive process: A study of discussion starters in the field of solar fuels

**DOI:** 10.1177/03063127241271024

**Published:** 2024-08-12

**Authors:** Eugen Octav Popa, Vincent Blok, Cornelius Schubert, Georgios Katsoukis

**Affiliations:** 1 Delft University of Technology, Delft, The Netherlands; 2 Wageningen University & Research, Wageningen, The Netherlands; 3 Technical University of Dortmund, Dortmund, Germany; 4 University of Twente, Enschede, The Netherlands

**Keywords:** Path creation, path dependence, discursive strategies, artificial photosynthesis

## Abstract

When a technology is seen as the right solution to a recognized problem, the development of alternative technologies comes under threat. To secure much-needed resources, proponents of alternative technologies must, in these conditions, restart societal discussion on the status quo, a process at once technological and discursive known as ‘path creation’. In this article, we investigate discussion-restarting strategies employed by supporters of emerging technologies in the field of solar fuels, particularly the advocates of a technology referred to as ‘artificial photosynthesis’. For illustrative purposes we explore four such strategies: revisiting weak spots, resizing the problem, redefining the game, and renegotiating labels. We conclude with a methodological reflection on the empirical study of discursive strategies in a socio-technical system. We further suggest a more systematic application of discourse-analytical and argumentation-theoretical insights that can complement current scholarship on path dependence and path creation.

To develop new technological solutions for recognized social needs, innovators compete with one another for resources like economic and human capital, favorable policies, public acceptance, materials, and space. During such a ‘battle for technological dominance’ (Suarez, 2004), a particular technology can come to dominate the field if stakeholders are satisfied with its ability to serve predetermined values and ideals (Bijker, 1997; Dao, 2019; Dosi & Nelson, 2010; Tang, 2006; Utterback & Suárez, 1993). A system’s loyalty towards technologies with a proven track record is sometimes referred to as *path dependence* (see, e.g., Aghion et al., 2019; Arthur, 1989; Berkhout, 2002; David, 1985) or as *technological lock-in* (Foxon, 2014; Rip & Kemp, 1998). In the context of limited resources dedicated to innovation, such success can be detrimental to those who proposed alternative technologies. In the case of large-scale systems, where start-up costs are high and positive feedback makes change difficult and expensive, the result is that ‘the more a technology is adopted, the more likely it is to be further adopted’ (Foxon, 2014, p. 305). In these conditions, while the winning solution is further optimized and the interest in alternative (‘losing’) technologies fades out, the assumption that the recognized problem is solved can become commonly accepted.

Yet technological lock-ins are sometimes broken. An established flow of resources can sometimes change, resulting in what is called *disruption* (Gobble, 2016; Millar et al., 2018) or *path creation* (Garud et al., 2010; Sydow et al., 2012). While the focus has primarily been on technology or system features conducive to change, more attention should be paid to the *discursive* dimension of these changes; the formation of ‘discourse coalitions’ and the sustained discursive negotiation of practices, norms, and storylines has been relatively neglected (see Hajer, 2002; Hess et al., 2010; Riedy, 2020). But to secure essential resources in a context of technological lock-in, proponents of alternative technologies that cannot yet be prototyped or demonstrated must ‘argue themselves into place’ (Gross, 2006, p. 43). When you cannot walk the walk, you can always talk the talk; stakeholders who want to change the socio-technical system must find ways to reopen the discussion on fundamental questions regarding the problem to be solved, the criteria for evaluating solutions, the hierarchy for the selected criteria, and so forth.

In this article, we illustrate an argumentation-theoretical analysis of several discursive strategies deployed by proponents of alternative technologies for restarting discussion on existing technologies for renewable fuel production. We focus on the contemporary debate around the replacement of fossil fuels with *solar fuels*. There are various alternative processes for producing hydrogen from renewable sources, some involving water splitting (thermolysis and photolysis), others involving the thermochemical processing of biomass (pyrolysis and gasification), and still others involving the biological processing of biomass (photo-fermentation). Each of these comes with its own techno-economical advantages and disadvantages (Nikolaidis & Poullikkas, 2017) as well as a specific socio-ethical moral profile of values served and disserved (Scott & Powells, 2020). More important for our purposes, however, is that most of these currently enjoy a lower level of technological readiness (TRL) and commercial deployment than the established solution. In our case study, we thus ask: How do proponents of alternative technologies re-open discussion on the established PV-e solution? We, therefore, seek to continue the growing interest in studies of path dependence and path creation in studying energy systems and their transition towards sustainable green technologies. Still, we would like to draw attention to the discursive dimension of the ‘battle for survival’, how technologies are argumentatively introduced, promoted, institutionalized, criticized, and eventually replaced (Bahn-Walkowiak & Wilts, 2017; Bolwig et al., 2019; Foxon et al., 2005; Groen et al., 2022).

## The discursive dimension of path creation

The positive reinforcement of established technologies, or ‘path dependence’, is occasionally interrupted by episodes of systemic change (Arthur, 1989; Garud et al., 2010; Sydow et al., 2009, 2012; Vergne & Durand, 2010). This dynamic of stabilization and destabilization has been thoroughly studied from behavioral, organizational, and system-analytical perspectives, yet the discursive dimension has generally been overlooked. For example, in an otherwise comprehensive and informative study of the obduracy of centralized infrastructural systems, Moss (2016) virtually ignores the discursive dimension of the interaction between opponents and proponents of entrenched technologies. Similarly, the most prominent theory of disruptive innovation pays little attention to the discursive process through which new actors argue themselves into place (Christenson, 1997; Si & Chen, 2020). Finally, the theory of path creation acknowledges that contingencies are ‘emergent’ and that the positive reinforcement mechanisms are ‘strategically manipulated by actors’, yet they do not detail the primarily discursive medium through which all this is achieved (Garud et al., 2010).

In science and technology studies, innovation and socio-technical change are often understood in discursive terms.^1^ For example, the discursive dimension receives attention in historical studies of how the ‘battle for survival’ is fought in various domains at specific historical junctures (Lynch et al., 2010; Schubert et al., 2013; Shapin & Schaffer, 2011). Similarly, Hess and colleagues insist that path dependence implies “the prevalence of certain speakers and storylines”, and that the discourse-analytical perspective can help uncover how actors are excluded from or, in case of change, *included in* the discourse surrounding a recognized socio-technical issue (Hess et al., 2010, p. 204). Goldstein and colleagues have noted that ‘the concept of feeling “trapped” into certain technologies, behaviors, and relations emerges across contexts and in public and policy discourse’ (Goldstein et al., 2023, p. 2). In the study of scientific discourse, apart from the technological question, paradigm change has traditionally been the topic of various discourse-oriented fields, such as argumentation studies (Rehg, 2008, 2009), rhetoric (Ceccarelli, 2001; Gross, 2006; Kirk & Kutchins, 2017), and controversy studies (Kleinman et al., 2005; van Eemeren & Garssen, 2008). The notion of *technological conflict* has also been employed to highlight the need for a more systematic and reflective incorporation of discourse-analytical techniques into the study of technological path creation (Popa et al., 2021).

In this paper, we want to illustrate an analytical standpoint that focuses on discussion moves, defined as goal-directed argumentative discourse (including, but not restricted to, actual arguments) deployed during a state of conflict. The notion resembles ‘argumentative moves’, but we do not see the need to restrict the interactants’ goal to resolving disagreement (van Eemeren & Snoeck Henkemans, 2016). We assume that stakeholders can entertain a variety of goals while engaged in technological conflict: they can aim to resolve their disagreement but also to prolong or postpone it; they can aim to work towards a compromise or towards an ultimatum or settlement. They can aim to expand or contract the scope and duration of the conflict, muddy the waters or clarify things, etc. The strategic relation between actors’ discourse and their goals, i.e., the *goal-directedness of discussion moves*, is often implicit. The analyst’s task is to make it explicit during discourse-analytical reconstruction by appealing to various forms of empirical evidence, from textual evidence of the interaction to the broader textual, inter-textual, and contextual evidence that can be brought to bear on the case at hand.

One category of meaningful discussion moves in the context of path creation is those through which actors seek to close or (re)open discussion on existing technological solutions (see Moss, 2016). Proponents of existing technologies should want to preserve the advantageous status quo (keep the discussion closed). In contrast, proponents of alternative technologies should like to challenge the status quo (reopen the discussion). Thus we distinguish *discussion stoppers* and *discussion starters.*

Two methodological notes: First, the categories of discussion starters and discussion stoppers are discourse-analytical ones, not moral or epistemic ones. In the past, discussion stoppers have had something of a bad reputation as either epistemic faults, such as Popper’s (1989) ‘immunization strategies’, or moral ones, such as Stirling’s (2008) *closing down* of ssocietal debates (see also Stilgoe et al., 2014). By contrast, there is a case for discussion starters’ moral and epistemic superiority, since they instantiate a more critical and democratic approach to technology governance (Cuppen et al., 2019; Popa et al., 2021). For the present purpose, we focus on *how* actors seek to restart discussion regarding established technologies to create a discursive space for their proposed alternative, but without adopting a normative stance.

Second, we do not place any restriction on participants, topics, mediums, durations, or intensities for those interactions between actors that can count as (as part of) the discussion on a particular technology. In this, we follow the discourse-coalition approach, where the discursive and non-discursive dimensions of a societal phenomenon intertwine without predetermined restrictions on where and how discourse coalitions form and interact (Hajer, 2002). Of course, for different analytical purposes, some interactions are more interesting than others. For example, a case may be interesting because the actors involved are closer to the policy-making process, or perhaps because they are far from it. However, these considerations are not intrinsic to the notion of discussion, and a full-blown analysis of the technological conflict around a specific technology should aim for a diversity of parameters. Technological conflict can be tackled in various settings and configurations: informal and anonymous online discussions, high-level policy discussions behind closed doors, academic publications, industry negotiations, media, etc. There is little basis to contend that one of these environments is by fiat more important or interesting than the other.

## Data and method of analysis

The term ‘solar fuel’ is used with some variation, though generally referring to cases in which solar energy drives chemical reactions. This process results in fuels that can be used as feedstock in industry or for heating and mobility (Nocera, 2017). Knowing that the Sun bombards our planet with significantly more energy than we would need to power the earth, the commonly expressed hope is that solar fuels could constitute a viable solution for replacing fossil fuels (Gray, 2009; Gust et al., 2009). The question, however, is how to produce such fuels.

There already exists an accepted technological solution. The ‘PV-e’ solution has a high TRL and is being commercialized worldwide. Solar energy is harvested through photovoltaic panels (‘PV’) and employed as electricity to drive electrolyzers (‘e’) that split water into hydrogen and oxygen. The hydrogen is subsequently used as feedstock in the industry or to synthesize more complex fuels, such as ammonia (NH3) (Chatenet et al., 2022). PV-e is, therefore, a combination of two relatively mature technologies applied in numerous sites across the globe.

Nevertheless, there is a continued discussion concerning this solution. Stakeholders from various fields have sought alternative pathways for a more direct (or more efficient) conversion into usable fuels. In this category, we include researchers who describe their work as the study of artificial photosynthesis (AP), a term that suggests a parallel with the natural photosynthetic process (Gust, 2016; House et al., 2015). Such technologies, however, are still in a phase of low-TRL fundamental research; it is unclear how to build such a device and, according to skeptics, whether such a device is even possible given resource conditions on Earth. We can thus identify the following two discourse coalitions:

**(i) Proponents of the established technology (PV-e)**
  - technologies already available (high TRL)
  - stakeholders seek resources for optimization and upscaling
  - electrolyzers can be combined with other renewable electricity sources, such as wind or hydropower
  - benefit from current resource flow (e.g., governmental subsidies)
  - established actor networks
**(ii) Proponents of alternative technologies (AP)**
  - technologies are only available in lab settings (low TRL)
  - stakeholders seek resources for development and prototyping
  - unclear how the technology combines with existing technologies
  - benefit less from the current resource flow
  - unestablished or small actor networks

Aside from AP, many other alternative ways of producing hydrogen are currently being explored, some of which do not seek to employ solar energy as a primary source (Nikolaidis & Poullikkas, 2017). Proponents of PV-e and AP share the ambition to find cost-effective means of harvesting solar energy and storing it within the chemical bonds of usable fuel. However, what differentiates them is their current position within the system and their current relationship with established technological pathways. Electrolysers are ‘already here’ and are increasingly employed for decarbonization, whereas the artificial leaf is ‘not here yet’ and needs resources for its development.

Our analysis focuses on how proponents of alternative technologies seek to restart the discussion on the established solution. We employ different sources of empirical data. First, we have had direct contact with proponents of alternative technologies occasioned by an ethnographic study of their community in The Netherlands between November 2021 and February 2023. In this period, we drew upon the tradition of studying scientists in their environment (Fisher, 2007; Johansson & Boholm, 2017; Knorr Cetina, 1995; Latour & Woolgar, 2013).

We distinguished between unprompted and prompted forms of participation in the conflict. Samples of unprompted participation were gathered from (i) academic articles written by proponents of alternative technologies within the expert community, (ii) oral communication during various community meetings, and (iii) oral communication in the lab and the workplace. Data from sources (ii) and (iii) were captured using field notes taken at the end of the interaction. In addition, two sources of prompted participation were employed for this study: (iv) semi-structured interviews with eleven proponents of alternative technologies and one PV-e proponent, and (v) impromptu verbal interaction at the workplace. Both were occasioned by an interdisciplinary research project in which social scientists and philosophers were given access to the workspace of natural scientists engaged in research on artificial photosynthesis at the University of Twente. In the next section, all referenced quotes are from the first source while all unreferenced quotes are from the last four. For reasons of anonymity, we cannot give any further information regarding the selected quotations from our fieldwork.

Given our concern with how proponents of alternative technologies seek to obtain visibility in a field dominated by more mature technologies, the analysis focuses on *discussion starters*, building on the idea that path dependence can be analysed in terms of its discursive dimension. Path dependence can be viewed as a societal discussion that was ‘won’ (or better: ‘continues to be won’) by actors supporting certain technological choices. Path creation can then be viewed as the re-opening of that ‘lost’ discussion. To further understand what discussion starters are and how they appear in technological conflict, we carried out a strategic reconstruction of the corpus described in Table 1. This involved analysing the participants’ discursive behavior as strategically directed towards identifiable discussion aims—in short, analysing contributions to the discussion as implementing one or more discussion strategies. In our case, we focus on the strategy of (re)opening the discussion. Following similar studies of discursive strategies (Hansson, 2015) and strategic maneuvering (van Eemeren & Houtlosser, 2006), we see it as beneficial to maintain a fruitful openness to the concept of ‘discussion starter’ to allow various micro- and macro-level discursive choices to count as instantiations. In the following section, we present four such discussion starters. Our aim is to illustrate both the concept of ‘discussion starter’ and the argumentative reconstruction required for identifying and understanding how actors seek to restart a discussion. Furthermore, we selected strategies that can presumably be identified in other technological conflicts since they are not technology-specific, i.e., they are not occasioned by the particular design parameters of specific technologies but rather by features that might be expected to arise in other instances of technological conflict.

**Table 1.** Selected corpus for studying discussion starters in the field of solar fuels.

Source	Description	Quantity
Unprompted participation		
Written communication	Academic articles within the field of (photo)electrochemistry that contain discussions of, or contributions to, the conflict	44 academic papers on artificial photosynthesis dated 2006-2023
Oral communication (community)	Oral communication during research seminars and conferences	3 research seminars, 1 international conference
Oral communication (workplace)	Oral communication between experts during lab activities and other activities at the workplace	~80 hours of observation and participation in workplace activities
Prompted participation		
Long-form interaction	Interviews with members of the newcomer’s community who are involved (directly or indirectly) in the development of conflict	12 semi-structured interviews 45-60 minutes
Short-form interaction	Impromptu discussions between the researchers and newcomers at the workplace inside and outside the lab	~80 hours of observation and participation in workplace activities

## Four illustrative discussion starters

The first discussion starter consists of revisiting the known disadvantages or ‘weak spots’ of the established technologies to justify the search for alternatives. The second consists of describing the scope of the recognized problem in such a way that one’s proposed technology fares better than the existing one(s) in these terms. The third consists of stipulating and assigning increased priority to a series of techno-moral requirements better served by one’s proposed technology. The fourth discussion starter consists of defining essential terms/expressions such that one’s proposed technology benefits from positive connotations and dissociates itself from negative ones. We will refer to PV-e technologies as the ‘established solution’ and to lower-TRL electrochemical technologies as the ‘alternative solutions.’ This is a language convention to avoid convoluted formulations and not an empirical description of how the actors themselves see their relationship with competitors. As we will see, whether PV-e should be seen as already established is itself up for discussion.

### Revisiting known weak spots

The first strategy identified in the corpus is revisiting weak spots, drawing attention to one or more limitations of the established technology. This is explicable in rhetorical terms because the positive image of an established solution is strategically disadvantageous for the proponents of alternative solutions. As the saying goes: ‘If it ain’t broke, don’t fix it!’

In the example below, taken from Thapper et al. (2013), a group of 16 researchers advances the case for the further study of artificial photosynthesis as an alternative solution for producing green fuels. The over-reliance on electricity is brought to the fore as a weak spot, first because most of our energy needs are not in the form of electricity—meaning that PV technology is of limited use without energy storage in fuel—and second because the upscaling of electricity is complex and can create infrastructural problems:

[I]t will, with time, become necessary to convert an increasingly larger share of renewable energy into gaseous or liquid fuels. One important reason is that most of the energy consumption in Europe (and globally) is not in the form of electricity …. Instead, more than 80% of the primary energy is used mainly as fuels to drive a wide variety of processes in our societies…A second reason is the limitations of the electricity grid, which prevents very large penetration of renewable electricity coming from intermittent energy sources often located far away from the final users. (Thapper et al., 2013, p. 45)

The paper further suggests that reliance on electricity is a liability even before considering the weak spots of the conversion process from electricity to fuels. Most of our energy needs can only be satisfied by fuels, while electricity grids constitute an infrastructural barrier.

The choice of what is criticized is essential. If the alternative solution cannot improve on the identified weak spots of the established solution (or worse, if the alternatives suffer from the same problem), then revisiting the weak spots seems irrelevant. After discussing the ‘electricity disadvantage’ of the established technology, the alternative technology, unsurprisingly, answers precisely these weak spots:

One promising solution to both these problems is Artificial Photosynthesis which provides all of the energy supply, the necessary energy storage and the facilitated transportation. In addition, Artificial Photosynthesis allows the utilization of renewable energy at both the local and the continental level, producing fuels made from solar energy and water (Thapper et al., 2013, p. 45)

Revisiting weak spots must, therefore, lead to the conclusion that the alternative technology fares significantly better on the selected account, i.e., that artificial photosynthesis is a ‘promising solution to both these problems.’ Proponents of the alternative solution need not be the ones who discover the weak spots for the first time. In our case, the fact that PV-e comes with disadvantages, given the use of intermittent energy sources and electricity infrastructure (cables, converters, etc.), is presented as a given. The problem is only posed, not discovered.

If the weak spot is known and accepted, proponents of the alternative technology must ensure that it is not minimized. For example, speaking on the PV-e solution, one of our interviewees insisted that the problems arising from the use of electricity are, in fact, impossible to solve under the assumption that the final goal is to produce cost-effective fuels. The weak spot in this case is represented as a limitation that cannot be overcome under the pressures of economic viability:

If you have too many Carnot cycles one after the other, losses will accumulate drastically ….You can leave it to the PV-electrolysis to solve the problem [of products recombining] but then you’re going to pay an enormous price … PV-electrolysis is scalable, you can make it as big as you want, but it will never be economically viable.

Given the low TRL of the alternative technologies, revisiting weak spots can backfire. If the established technology fares poorly in one regard, alternative technologies might fare even worse. Consider cost-effectiveness: The established technology can be attacked for being expensive, incurring costs that can only be justified by referencing some shared societal goal (e.g., fighting climate change or geopolitical independence). Yet the alternative technology might be even more expensive, at least now, given its low-TRL status and the lack of immediate opportunities for price reduction through optimization and upscaling. Under these conditions, proponents of the alternative technology must discuss weak spots not in terms of present conditions but rather in terms of potential achievements in the future. We see this in the quote above. The problem is not the current price of fuel made through PV-e, either in absolute terms or relative to AP, but rather the projected difference in cost after further development of both technologies.

Restarting the discussion on such forward-looking terms as how the technologies might fare on a chosen criterion in the future brings an additional strategic advantage. The high TRL of the established technology, usually a favorable argument for adopting a technology, suddenly appears as a liability. If a mature technology fails to meet our expectations of cost-effectiveness even after enjoying the benefits of optimization and upscaling, an unexpected and radical improvement on that front in the future becomes unlikely. Ardo et al. note:

On the cost side, only minor reductions are expected from silicon manufacturing [employed in PV panels], as the prices have already decreased significantly (currently at <USD 0.5 W^−1^) and gains from economies of scale will saturate. (Ardo et al., 2018, p. 2788)

There is always some probability that alternative, unexpected materials can result in surprising benefits, but the same authors are quick to cast doubt on such developments.

There are many factors that limit the practicality of each alternative PV material, such as stability, toxicity, efficiency, and durability, but ultimately, each of these technologies suffers from the same limiting factor for large-scale viability: economic competitiveness. (Ardo et al., 2018, p. 2788)

This opens a door for alternative technologies. Their youth, far from being a disadvantage, can be portrayed as a great promise. This explains why some interviewed proponents of the established technology rejected terms such as ‘established,’ ‘incumbent,’ ‘accepted,’ and even ‘existing’ or ‘current’. They insisted that connotations of completeness (of peak performance) are incorrect. Both sides of the PV-e marriage—the conversion of solar energy through photovoltaic panels and the conversion of electricity through electrolysis—must be seen as young, promising technologies even though they are already commercially available worldwide. It is the previous fossil-based technologies for fuel production that must be seen as the traditional path.

Weak spots are seldom advanced in isolation. Unless a case is made that the identified weak spot is a deal-breaker, proponents of alternative technologies will generally revisit multiple weak spots simultaneously. This is sometimes referred to as *coordinative argumentation* in that none of the presented reasons sufficiently support the standpoint on their own but together make a compelling case (Snoeck Henkemans, 2000). The high cost of fuel might be accepted in light of a recognized need to decarbonize various industries, but the image of the established technology will be affected more if this weak spot is combined with others, such as dependence on rare materials, geopolitical risks, infrastructural bottlenecks, and hard limits on upscaling. The alternative technology is relatively safe from such harsh ‘in-bulk’ examinations of defects (due to its low TRL, see previous point) and need not even score better on all the evaluation criteria brought in the discussion. Revisiting weak spots is not a means to win the discussion; it is a means to reopen it, to create a rhetorical space for alternative value propositions. The strategy’s effectiveness need not depend on the new technology’s perceived or actual ability to fare better on all the highlighted points, but on whether discussants can dispel the notion that the problem is already solved and that everyone is content with the present solution. However, whether the established technology solves the recognized problem will depend to some extent on the formulation of the problem.

### Resizing the problem

We are accustomed to ascribing ‘interpretive flexibility’ to technologies or, more generally, to artifacts (Latour & Woolgar, 2013; Pinch & Bijker, 1984). But the problem at hand is also interpretively flexible, especially for wide-ranging problems that involve entire societies or cultures. This opens up a possibility for proponents of alternative technologies to restart the discussion on existing technological solutions. Here, we want to focus on the strategy of *resizing the problem*, by which we mean changing the scope of the problem either spatially (the region and population that is confronted with the problem) or chronologically (the timescale for how long the problem has been present and how soon it needs to be solved). The proposed changes in scope need not occur through explicit declarative statements. Instead, they can remain implicit, building strategically on a shared idiom of labels, expressions, and commonplaces (*topoi*) that allow the interpretive exercise.

In our case, the dependence on fossil fuels is widely recognized as the problem at hand. Yet the scale at which the problem must be approached and the resulting timeline of change is up for discussion. Some problems are best approached through small, regional solutions that are developed piecemeal, while others require holistic, global, or radical solutions. Whether the proposed technologies must replace fossil fuels at a local, regional, national, or global level becomes an important question or, at the very least, a non-trivial one. If the case can be made that the established technology might not manage to solve the problem at the desired scale but only alleviate it somewhat, or solve it only partially for a smaller group, it can be strategic for newcomers to insist on the sheer size of the problem. The more a problem appears unsolvable by existing means, the more natural it becomes to restart the discussion on alternatives.

As expected, no explicit statements have been found in the corpus to the effect that we should look for big (global) solutions that match the size of the problem. However, newcomers often make clear in various ways that there is a discrepancy between the size of the recognized problem and the level at which established technologies solve or alleviate matters. Newcomers typically insist that solutions need to be applicable at a ‘terawatt scale’ (Messinger et al., 2018), that we are dealing with a ‘terawatt challenge’ (Đokić & Soo, 2018; Nocera, 2012b), and that we are standing before a ‘grand scientific challenge’ (Thapper et al., 2013). For example:

[L]et’s be absolutely clear, you first of all need 5 to 10 terawatt of energy dense … high energy dense transportation fuel. You can’t get around that, you know? You can’t fly an airplane with batteries, that’s not possible. You can make it with green hydrogen, but it has to be green .… Now when you ask the question ‘Can we make green hydrogen on a level of 5 to 10 terawatt with electrolyser and PV?’ I’m convinced, and others are also convinced that no, that’s not possible. The scale is too big!

A spatial resizing of this kind, through which a problem is portrayed as too big for the established solutions to handle, is vulnerable from a strategic point of view. Technologies that are by themselves only capable of relatively small impacts might, after all, manage to solve a global problem through multiplication and upscaling. A bigger problem might benefit proponents of the established technology by further deepening current path dependency. For resizing to work, proponents of alternative technologies must portray it as highly improbable that the established technology will ever tackle problems at the given (global) scale. A simple comparison between the current achievements of the established technology and the task at hand can serve to make this knife-to-a-gunfight argument. For example:

At this point, you would think that we have an immense amount of PV panels around the world. … It’s 0.35 terawatt! We have been working for decades and [unintelligible] ramping up, but we are not even at half a terawatt. It’s very difficult even for scientists to get the mindset of what scalability actually means. Terawatt is a frightening number. So if you have a 2 gigawatt plant, that’s huge. But that’s only 0.02 percent of a terawatt! [laughs]

Another way to make the same argument is to explain why the established technology should not be expected to meet these high expectations, or why it is inadequate for the task. In the quote below, we italicized the terms that refer to precisely those weak spots that hinder an application of the technology at a terawatt scale:

These criteria [i.e., large-scale production of cost-effective energy] are precisely the antithesis of the design and production of most energy systems of the legacy world. … Balance of system costs *do not scale* commensurately. Thus, off-the-shelf technology and ‘existing’ technologies will be difficult to adapt to low-cost energy systems. Simply put, new R&D is needed to provide our society with the ‘fast food’ equivalent of energy systems. Such *infrastructure is not viable* in the near-term future of nonlegacy states, where it is cost prohibitive to build centralized energy and distribution systems. (Nocera, 2017, p. 998)

By comparing how the technology fares in both the legacy and non-legacy worlds, Nocera implicitly suggests that the technology’s evaluation must occur globally and include both worlds. Then, to make the case that current technologies are unsatisfactory from this (spatial) perspective, he explains their failure by appealing to ideas such as balance-of-system and infrastructure dependence. Since he refrains from further explaining why these barriers cannot be overcome, we are invited to see them as a matter of accepted common ground.

Like the previous discussion starter, resizing the problem can be undertaken even when it is uncertain that alternative technologies will ever become more efficient than established ones on the desired scale. The knife-to-a-gunfight argument is only meant to show that the established technology fails to address the problem fully in all its magnitude. This alone can justify the allocation of resources for alternative technologies. Although we did not encounter this in our corpus, resizing the problem can also occur as a form of minimization, for instance, when alternative technologies offer tailor-made and local solutions, whereas the established technologies approach the problem globally and indiscriminately.

### Redefining the game

Does the established technology offer an acceptable solution to the problem of fossil fuel dependence? The answer will depend on the criteria that are in place for assessing proposed solutions. Once candidates have been shortlisted by this initial theoretical calculation, additional criteria must be established for evaluating proposed technological solutions. This dynamic process of institutionalization opens up a space for dialogue and negotiation among different discourse coalitions (Hajer, 2002). Proponents of alternative technologies can adapt their communication to this aim by advocating for or against specific evaluation criteria, setting or resetting the rules of the game to their advantage.

In the context under analysis here, where an established technology is already seen as an acceptable solution to the recognized problem, proponents of alternative technologies often advocate adding new criteria to the evaluation game. In this way, the perceived superiority of the established technology can be dissipated since, if the evaluation can be made stricter than it currently is, the winner might turn out not to be a true winner after all. In some cases, actors might suggest the more radical view that a full (or ‘true’) evaluation has yet to take place. The clearest examples of this strategy come from discussions of the material conditions in which the solution needs to be built, e.g.,

For the production of solar fuel to be economically and environmentally attractive, the fuels must be formed from abundant, inexpensive raw materials such as water and carbon dioxide. (Balzani et al., 2008, p. 32)

[It] will probably involve inexpensive and self-repairing components that operate at neutral pH with non-pure (salty or bacterially and chemically contaminated) water (Faunce, 2012, p. 353)

The illocutionary force of the speech acts in both quotes, the declaratives in the former and the assertives in the latter, is quite unclear. These are surely normative claims about what should be done, but the authors’ communicative intentions appear to be halfway between statements of fact and stipulations of game rules for acceptability. Proposed rules are often presented as ineluctable laws, as *shoulds* and *must nots*, but they are in fact rooted in normative considerations of desired efficiency, cost, scalability, system integration, and more generally in a perceived ideal future of the energy system. For example:

[T]he catalyst must be stable as long as possible to be economically feasible. Furthermore, the activity of the catalyst should be high to make use of all electrons provided by the sensitizer. Moreover, the hydrogen produced should not inhibit the catalyst in order to maintain a constantly high hydrogen evolution rate. (Krassen et al., 2011, p. 51)

[A]ssume you use Silicon as the light absorbing element. That’s in any case not enough because you need…because you don’t have enough voltage to split water. So, you still need a second semiconductor.

[W]e should forget about hydrogen because we cannot compete with the production of hydrogen from an electrolyzer

The first quote is part of a section titled ‘Criteria for “good” catalysts’. The authors’ choice to use scare quotes for the term ‘good’ is not incidental, but is a genuine recognition of the societal values required to evaluate catalysts for fuel production. Judging whether a catalyst is ‘good’ or ‘bad’ is as much a question of science and engineering as it is one of value-driven practical reasoning (Zwart et al., 2018). When such clear indicators of societal normativity are absent, it is often difficult to distinguish between hard limitations derived from the inescapable laws of physics and soft limitations derived from societal values. Consider:

[H]eterogeneous catalysts are generally preferred over homogeneous ones for benefits such as durability, ease of separation and recycling, among others. (Messinger et al., 2018)

Although the content is relatively clear, the illocutionary force of this statement is unclear. Is this a description of observable facts about what is generally preferred in a certain community? Is it a description of scientific fact regarding the ability of the two catalytic processes to meet the mentioned criteria? Whatever the case, we are presented with the rules of the game in the form of criteria that are accepted as valid and need to be satisfied.

Finally, since we are analying discourse around an alternative technology, it is essential for the proponents of this technology to stipulate what is possible or conceivable. Here as well, the line between statement of fact and stipulation of rule is ambiguous and provides space for strategic argumentation. Proponents of the alternative technology often insist that their technology is possible and is, in fact, already showcased in nature. After all, as the name suggests, *artificial* photosynthesis is nothing but a replication of existing *natural* photosynthesis. For example:

Photosynthesis traps 100-TW solar energy annually into biomass on land at 0.1% efficiency that is about six times more than global yearly energy demand …. The rampant rise in energy demand requires replicating the natural photosynthesis process artificially (Abas et al., 2020)

Photosynthesis is the largest-scale, best-tested method for solar energy harvesting on the planet. … The photosynthetic blueprint works, as indeed it must, because biology and technology are ruled by the same natural laws (Gust et al., 2009)

Nature has provided a blueprint and inspiration for averting and overcoming these energy and pollution crises (Đokić & Soo, 2018)

To highlight that natural photosynthesis can be replicated, newcomers often refer to it as nothing but a ‘challenge’. A challenge can be difficult but is attainable in principle and is, in any case, different from a vague ‘idea’ or an overly optimistic ‘hope’. Consider:

We know that it works. … Nature does it. The challenge is to replicate it!

It is still a great challenge to construct [a] highly efficient and robust artificial photosynthesis system for future scale-up application. Therefore, new catalyst materials and strategies for efficient OER are still imperatively required. (Ye et al., 2019, p. 52)

Far from being an ideal, natural photosynthesis is a proof of concept and, as such, a determination of what must be accepted as possible within the game. It is possible to convert solar energy into usable fuels because ‘Nature’ accomplishes just that through natural photosynthesis. Combined with the mentioned theoretical calculation of the oversupply of solar energy bombarding our planet every year, the connection between AP and its natural counterpart becomes an invaluable discursive resource.

Redefining the rules of the game is a discussion starter by means of which proponents of alternative technologies seek to revise the criteria through which the acceptability of technological solutions is evaluated. As we have seen, the entire deontic spectrum can be revisited, from that which is forbidden (by natural or moral laws), to the preferable (given accepted or posited criteria), and the possible (given lessons from Nature).

### Renegotiating semantics

*Both proponents for* the established technology and the alternatives will seek to associate their choice with words and expressions that carry a positive connotation. These positive connotations hardly need mentioning: technologies should be reliable, safe, efficient, just, cheap, and so forth, criteria that are typically built into technologies through their design (Van den Hoven et al., 2012). But how this valuation applies in a specific context and what positive connotations are to be gained provides some space for maneuvering. In our case, the established technology has laid claim to the idea of being ‘green’. Photovoltaic panels provide ‘green’ electricity and hydrogen made through PV-e is generally referred to as ‘green’ (and sometimes ‘renewable’) hydrogen.^2^ The positive connotations of the adjective ‘green’—sustainability, naturalness, and freshness—are hereby absorbed by the established technology. Although some have sought to use the term ‘golden hydrogen’ for solar fuels (Lubbe et al., 2022), proponents of alternative technologies have so far directed their efforts towards other terms carrying positive connotations.

First, the terms ‘artificial photosynthesis’ and ‘artificial leaf’ are semantically associated with their natural counterparts. Nature not only provides a blueprint for technological development, but a moral backing for the value of technology. What counts as artificial photosynthesis has yet to be decided. It is not unusual, we are told, for PV-e to be described as a form of artificial photosynthesis—although the more specific idea of an ‘artificial leaf’ remains associated exclusively with alternative technologies for photo(electro)catalysis (Bensaid et al., 2012; Nocera, 2012a; Zhang et al., 2021). In early papers on artificial photosynthesis, and sporadically in more recent publications, this broad use of the term includes ‘any man-mediated process which stores sunlight energy in useful, high energy chemicals’ (Bard & Fox, 1995; Bozal-Ginesta & Durrant, 2019). This ambivalence is apparent in the following dialogue between an interviewer and a respondent.

I: (mentions artificial photosynthesis, announcing the theme of the interview)

R: I never use that term. That’s the honest answer. Because when I think of artificial photosynthesis I’m thinking more of a kind of leaf. At least, I think first of normal photosynthesis, a leaf in a tree, so I’m thinking of something biochemical. That’s what I’m thinking of. But then when I thought about it more, everything that converts solar energy into chemical energy could be categorized like that and in this way it includes more. So the solar panel connected to electrolyser is also…

I: A kind of…

R: Artificial photosynthesis, you could say. So that’s the…

I: And if you don’t use this term, which one do you use instead? Solar fuels or green fuels?

R: I talk about hydrogen. Just hydrogen. That is my work. Hydrogen in fact. All kinds. Electrolysis. I’m an electrochemist so I concentrate not on how solar energy is converted but how you take electricity to make hydrogen…

There is thus a danger that the established technology exploits not only the rhetorical benefits of the label ‘green (hydrogen)’, but also the ones associated with artificial photosynthesis’. Proponents of alternative technologies must, therefore, insist that the label AP does not apply correctly to the established technologies. Among the arguments advanced to this effect are that the established technology is too ‘roundabout’ or ‘indirect’ or, as some scientists put it, ‘the long way around’ (e.g., Krassen et al., 2011). Yet it seems strategically easier to broaden a term than to restrict its application to a handful of technologies, so a restricted semantics always needs additional justification. Consider how the following interviewee started with a very broad definition of artificial photosynthesis and then proceeded to criticize it:

Artificial photosynthesis is the translation of natural photosynthesis to artificial processes, so inspired by nature—what happens in plants—but replicated and maybe even a bit improved. So you take sunlight and convert it *to something other than electricity*, to fuels and chemical products. Artificial photosynthesis has become a bit of an umbrella term that is used for something more than what I just described—so also PV plus electrolysis and photoelectrochemical cells.

It is important to note that even if the term ‘artificial photosynthesis’ is restricted to some of the alternative technologies, it can also serve to obviate disciplinary differences between separate approaches. In the beginning phases of technology development, this uniformity can be used strategically in assertions regarding, e.g., increased attention to the field of artificial photosynthesis. Bracketing the differences between alternative methods for carrying out artificial photosynthesis can thus be beneficial in providing an impression of a living—or perhaps ‘booming’—field of research.

Aside from the actual name of the technology, other terms and expressions can play a similar role in suggesting the need to reopen the discussion on the established technology. Another telling example, in this case, is the use of the label ‘the Holy Grail’. The label was particularly common in our selected corpus, suggesting that it helps proponents differentiate themselves and simultaneously add weight to the suggestion that their search is justified (Abas et al., 2020; Bard & Fox, 1995; Brinkert, 2018; Canter, 2022; Đokić & Soo, 2018; Luo et al., 2019; Wu et al., 2018). Is the Holy Grail something you self-evidently want to find? While the term ‘artificial photosynthesis’ brings with it the risk of being ‘hijacked’, ‘the Holy Grail’ is clearly protected against such hijacking. A certain technology can only be ‘the Holy Grail’ if it has yet to be found (as is the case with the Holy Grail), meaning that the established technology cannot fit the bill. An analogous case can be made for the label ‘fast food energy’—a metaphor for cheap energy that implicitly undermines the relatively high cost of hydrogen resulting from the established PV-e solution field (Nocera, 2010, 2012b, 2017).

## Towards a discourse analysis of path creation

We have illustrated a discourse-analytical approach to path creation in an asymmetric technological conflict. The conflict is triggered by resource scarcity and, as we hope to have shown, demands a certain level of argumentative creativity on the part of proponents. It is not easy to reopen a closed discussion. Like a chess player who loses the center of the board during the opening and must develop strategic options on the flanks, proponents of alternative technologies must dislodge a consensus that is disadvantageous to them by (1) revisiting weak spots, (2) resizing the problem, (3) redefining the game and (4) renegotiating semantics. This creative maneuvering can provide a better understanding of path dynamics in a socio-technical system and can be a source of inspiration for the development and maintenance of societal discussions on new and emerging technologies (Blok, 2019; Guston et al., 2014; Mouffe, 2013). We have suggested that analysing the discursive participation of defenders of different technologies is essential to understanding the dynamics of socio-technical change. In this concluding section, we would like to make several methodological observations as contributions to a more sustained study of the discursive dimension of path dependence and path creation.

Let us start with the obvious remark that the four strategies discussed here are only a few that could be employed to restart the discussion on established technologies. We have selected them for their salience and the variety of strategic considerations they involve. Although we suspect that they are not exclusive to the field of solar fuels, we have not attempted to demonstrate their broader applicability. Variation must be expected because technological lock-ins come in degrees, and different fields will lend themselves naturally to some strategies and not others.

A second observation concerns our operationalization of the distinction between proponents of the established technology and those of alternative technologies. At the beginning of our fieldwork, we had few reasons to doubt this distinction between the two groups. Since both photovoltaics and electrolysers have a high TRL and are already commercialized across the globe, while artificial leaves are barely at the prototyping stage (which incidentally also holds for many other hydrogen production methods), the established/alternative distinction seemed obvious. However, looking back on some of our interviews we realized that the PV-e solution is, in fact, an alternative technology (relative to the established fossil-based ones), so our operationalization was hardly self-evident. The fossil fuel industry can count as the established technology, rather than the relatively newer PV-e solutions. Indeed, the fact that we interviewed one isolated respondent from the PV-e camp was a happy mistake resulting from this ambiguity.

Even within the AP community, questions of scholarly delineation allow for different answers based on what method is seen as more developed or more established. Many studies of AP trace their history back to a breakthrough discovery of the light-driven water splitting through direct bandgap excitation of titanium oxide, yet various groups now belonging to the AP community work on fundamentally different approaches (Fujishima & Honda, 1972). The same holds, as one might expect, for the established technology. One could argue that there are no such things as electrolysers *simpliciter—*no ‘e’ in ‘PV-e’—but only different approaches to doing electrolysis, which result in technologies with different properties and levels of readiness, such as PEM electrolysers, alkaline electrolysers, solid oxide electrolysers, etc. When we look at two different ways of doing electrolysis, are we looking at two technologies or two different artifacts instantiating the same technology? The socio-technical system may be carved up into distinct technologies for analytical purposes such as ours, but there is generally, and perhaps always, some degree of artificiality to such distinctions. This is relevant for the analysis of technological conflict because, in analysing discourse, scholars generally work with a clear-cut distinction between opposing sides. One could argue that there is a conceptual tension between the technology readiness scale and the often-used distinctions between incumbents and newcomers (or, for that matter, the argumentation-theoretical distinction between proponents and opponents and between statements and their negation).

Finally, we wish to insist on the necessity of blending discourse-analytical and system- and organization-analytical perspectives in the study of path creation. Although, as noted in Section 1, the discursive dimension has generally been ignored or covered only indirectly in studies of the ‘social construction of facts’, both the discursive and the non-discursive approaches are needed for a full picture. Participants in societal discussion on solar fuels are doing things, both with words and without words, to use Austin’s (1962) phrase. Participants are constantly engaged in arguing, defining, explaining, stipulating, etc., but also in designing, building, testing, organizing, pushing, moving, activating, gathering, recording, etc. Technologies, seen as embodied techniques that acquire meaning only insofar as they appear within established forms of life, must therefore be seen as interwoven with discourse or even as forms of (performative) discourse in their own right (Coeckelbergh & Funk 2018). For example, an engineer’s decision to build an experimental AP cell using only common materials is, at the same time, a non-discursive move explainable by reference to the physical properties of these materials, and a discursive one explainable by reference to societal demands for technologies that only use abundant materials. Seen through argumentation-theoretical lenses, the choice to work with, say, copper and silicone will appear as simultaneously a technical and dialectical decision, the dialectical aspects relating to the potential counterarguments that one’s technology can never work without rare materials. Put differently, the case for AP would be significantly weakened if researchers were working exclusively with the same scarce materials used for the established technology. We insist, therefore, that a full understanding of technological conflict requires a blending of discourse-analytical and system- and organization-analytical perspectives.

## Notes

1. Of course, non-disruptive innovations also exhibit this argumentative dimension. The fifth razor blade, the thinner TV, the lighter bike—all these must be argued into place. But this goes without saying in the minds of both researchers and practitioners. It is ironic, then, that we are receptive to argumentation when the conflict is shallow and pertains only to a small set of assumptions about a product, but tend to overlook it when the conflict touches upon fundamental assumptions of the status quo. It is almost as if there is a limit to how much systemic change we are ready to explain by means of stakeholders’ argumentative behavior.

2. It is worth noting that the present paper was written in a context where the exact conditions for calling hydrogen ‘green’ are under intense discussion and lobbying both within the European and US markets. While US regulation is still unclear, two EU Delegated Acts specify numerous requirements such as additionality, temporal, and geographical correlation (Collins& Martin, 2023 ).
